# The Regulatory Role of MicroRNAs in Obesity and Obesity-Derived Ailments

**DOI:** 10.3390/genes14112070

**Published:** 2023-11-13

**Authors:** Javier A. Benavides-Aguilar, Andrea Torres-Copado, José Isidoro-Sánchez, Surajit Pathak, Asim K. Duttaroy, Antara Banerjee, Sujay Paul

**Affiliations:** 1 Technical University of Denmark, 2800 Kongens Lyngby, Denmark; 2 NatProLab-Plant Innovation Lab, School of Engineering and Sciences, Tecnologico de Monterrey, Queretaro 76130, Mexico; 3 Chettinad Academy of Research and Education (CARE), Chettinad Hospital and Research Institute (CHRI), Department of Medical Biotechnology, Faculty of Allied Health Sciences, Chennai 603103, India; 4 Department of Nutrition, Institute of Basic Medical Sciences, Faculty of Medicine, University of Oslo, P.O. Box 1046, N-0316 Oslo, Norway

**Keywords:** miRNA, obesity, adipogenesis, lipogenesis, adipose tissue

## Abstract

Obesity is a condition that is characterized by the presence of excessive adipose tissue in the body. Obesity has become one of the main health concerns worldwide since it can lead to other chronic ailments, such as type 2 diabetes or fatty liver disease, and it could be an aggravating factor in infections. MicroRNAs (miRNAs) are small, non-coding RNA molecules that regulate gene expression and can play an important role in controlling crucial biological processes involved in the onset of obesity, such as lipogenesis, adipogenesis, lipid metabolism, or the regulation of cytokines and chemokines. Moreover, chemical compounds present in food or food packaging can alter miRNA expression and regulate the aforementioned biological mechanisms related to diabetes onset and progression. Furthermore, therapies, such as bariatric surgery and aerobic exercise training, can also influence the expression profile of miRNAs in obesity. Therefore, the present review provides insight into the current research on the role of miRNAs in obesity and obesity-derived ailments, intending to develop novel therapies to effectively manage these disorders.

## 1. Introduction

Obesity is a condition in which adipose tissue (AT) mass is present in excess in the body. An individual is considered obese when their body mass index (BMI) is over 30 kg/m^2^ [1]. Obesity prevalence has increased alarmingly in recent decades, mostly due to consuming unhealthy, energy-dense foods and sedentarism [2].

It is estimated that currently, over 2 billion people are suffering from obesity worldwide, and in consequence, they possess a high risk of developing several obesity-associated metabolic disorders, such as type 2 diabetes (T2D), fatty liver, hypertension, heart failure, thrombosis, sleep apnea, physical impairment, several types of cancer, and even mental health disorders such as depression or anxiety [2,3]. Obesity is also a crucial comorbidity factor in infectious diseases, such as influenza A H1N1 or COVID-19, as seen in the 2009 and 2020 pandemics [4]. Hence, obesity is a world health concern that is having a deep financial burden on public health systems by spending, for example, more than $190 billion US dollars annually in order to tackle obesity-related ailments [3].

Body mass augments when the energy expenditure is lower than the energy consumption; hence, a positive energy balance will lead to obesity consequently [5]. Two main systems control food intake: the hedonic and homeostatic pathways. The hedonic (or reward-based) pathway supports energy homeostasis when there is a period of energy deficiency by mediating the motivational side of food consumption. On the other hand, the homeostatic pathway stimulates eating behavior, with the brainstem and hypothalamus at their centers. In the homeostatic pathway, feelings of hunger and satiety are mediated by integrating central and peripheral signals, including gastrointestinal hormones. Along with the main food intake regulatory systems, neurotransmitters like serotonin or dopamine are closely related to obesity development [5,6].

Moreover, several genetic causes can trigger the pathogenesis of obesity, and genetic abnormalities account for up to 70% of severe, early-onset obesity [7]. There are three main genetic causes of obesity: monogenic, syndromic, and polygenic [8]. Monogenic obesity is caused by variants of only one gene [9], and several genes have been associated with this type of obesity, such as leptin (LEP), leptin receptor (LEPR), pro-opiomelanocortin (POMC), and melanocortin 4 receptor gene (MC4R), which is the most common form of monogenic obesity [10]. Syndromic obesity is a rare-occurring type of obesity, and it is often related to various phenotypes, including dysmorphic features, organ-specific abnormalities, intellectual disabilities, or developmental delay [11]. Finally, polygenic obesity, also known as common obesity, results from several factors, such as multiple polymorphisms, an interaction of obesogenic environments, and other genetic variables [12]. Moreover, epigenetic factors also contribute to the development of obesity, relating environmental factors to patterns of genetic change [5,13]. The most relevant epigenetic mechanisms involved in obesity are DNA methylation, histone modifications, and microRNAs (miRNAs) [13].

miRNAs are small, non-coding RNA molecules (20–24 nucleotides) that are involved in numerous biological processes [14,15]. These tiny molecules are highly conserved and act as regulators of gene expression through the attenuation or degradation of messenger RNA (mRNA) [16,17]. Modulation of gene expression occurs when miRNA matches with its complementary sequences in the 3′ untranslated region (3′-UTR) of the target mRNA, resulting in mRNA degradation or translation repression [18,19]. Hence, crucial cellular processes, such as apoptosis, cell growth, proliferation, and differentiation, are regulated by the expression of miRNAs [20,21,22,23]. Biogenesis of miRNAs occurs mostly by the canonical pathway, which initiates with the transcription of primary miRNA (pri-miRNA) transcripts [24,25]. Subsequently, the Drosha complex, formed by the DGCR8 protein and the ribonuclease (RNase) III enzyme Drosha, processes the pri-miRNA into precursor miRNA (pre-miRNA). Afterward, Exportin-5 (XPO5) exports the pre-miRNA from the nucleus to the cytoplasm, where an RNase III called Dicer further processes the pre-miRNA into a 21–24 nucleotide miRNA/miRNA* duplex. Consequently, chaperone proteins such as HSP70/HSP90 load the newly formed duplex into an Argonaute (Ago) protein in order to build the RNA-induced silencing complex (RISC). Only one of the two strands (the mature miRNA) is retained in the complex, and the other strand, known as the miRNA*, is disposed of. To induce target repression, the RISC complex binds to the 3′UTR region of the target mRNA [24,25,26,27].

miRNAs are substantially involved in the pathogenesis of multiple chronic diseases, such as cancer [14,28,29], diabetes [30], endocrine disorders [17], and obesity [31], and could contribute to a better prognosis and management of those ailments [20].

**Figure 1:**
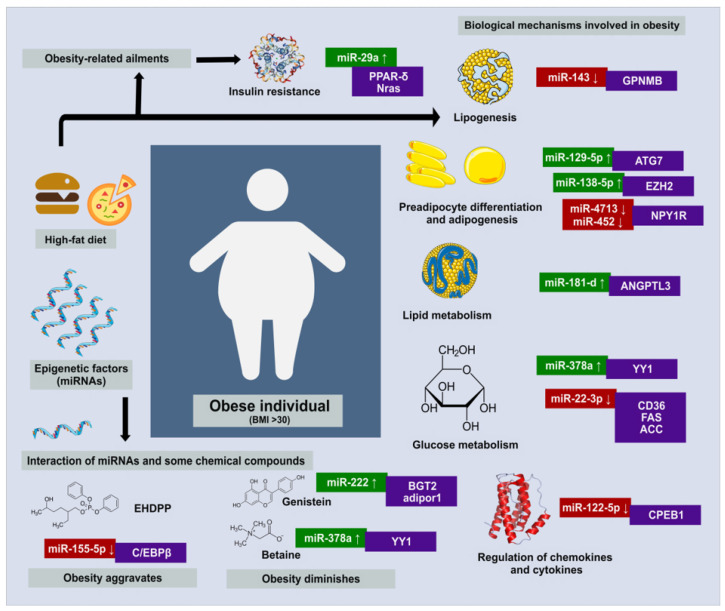
Role of miRNAs in the development of obesity and related ailments. One of the main causes of obesity is a high-fat diet, leading to lipogenesis and an augmentation in AT mass and weight. Moreover, chemical compounds in food or food packaging can exert a beneficial or detrimental effect on obesity onset. A number of biological mechanisms are involved in obesity development, and some of them are regulated by different miRNAs and their corresponding targets, as shown in the figure. Relevant miRNAs involved in the presented metabolic pathways are only partially listed. Green boxes indicate miRNA upregulation, and red boxes indicate miRNA downregulation.

Although a number of biological mechanisms are involved in obesity development, several of them, such as lipid metabolism [32], adipogenesis [33], glycolysis, gluconeogenesis, thermogenesis, and regulation of cytokines [34], could be controlled by miRNAs (Figure 1). Hence, elucidating the precise regulatory role of miRNAs in obesity pathogenesis is relevant in developing novel strategies to combat this disorder (Table 1).

## 2. Roles of microRNAs in Obesity

Several miRNAs have been associated with the development of obesity, including processes crucial for pathogenesis, such as adipocyte differentiation. Fu et al. (2019) [40] conducted an in vivo study to establish the relationship between miR-129-5p and adipocyte differentiation. They found that in the epididymal white adipose tissues (EWAT) of obese mouse models, miR-129-5p was significantly overexpressed. Moreover, upregulation of miR-129-5p inhibited white fat tissue differentiation as well as the expression of several crucial genes involved in adipogenesis, like C/EBPα, ABP4, PPARγ, and FAS. Additionally, higher miR-129-5p levels suppressed beige adipogenesis and diminished the expression of UCP1, PRDM16, and PPARγ, which are adipogenic genes and precise markers of brown mature adipocytes. Interestingly, ATG7, an essential autophagy gene, is directly targeted by miR-129-5p; thus, the upregulation of this miRNA could inhibit autophagy; nonetheless, the detailed mechanism of adipocyte differentiation via the ATG7-related autophagy pathway needs further investigation. The authors also measured miR-129-5p levels in the serum of 16 patients with simple obesity. Consistent with the results obtained in the mouse models, miR-129-5p was overexpressed in obese patients. Furthermore, they found a positive correlation between obesity rates, such as BMI and fat percentage, and miR-129-5p expression, indicating that circulating miR-129-5p could be used as an obesity biomarker. Taken together, miR-129-5p could be a potent candidate for developing miRNA-based advanced therapeutic strategies against obesity [40].

Abu-Farha et al. (2019) [32] investigated miR-181d expression in the blood of AT in a cohort of obese and non-obese people and assessed whether miR-181d can bind and regulate ANGPTL3 in hepatocyte cell cultures. They found that miR-181d levels in plasma were significantly lower in obese people than non-obese ones. Additionally, in vitro analysis confirmed that miR-181d exhibited selective binding and repression of ANGPTL3 [32]. Plasma levels of miR-181d showed an inverse correlation with triglyceride (TG) levels, while ANGPTL3 levels positively correlated with TG levels. In obese individuals, miR-181d expression is significantly reduced in both blood and AT. Evidence suggests that miR-181d plays a protective role against obesity by regulating lipid metabolism and could potentially be targeted for therapeutic interventions in managing dysregulated lipid metabolism in metabolic disorders.

The differentiation of preadipocytes (precursor cells to adipocytes), the main component of fatty tissue, can be regulated by the interaction of miRNAs and circular RNAs (circ-RNA) [33,36]. In a recent study, Liu, Y., and colleagues (2020) overexpressed miR-138-5p mimics in AT samples to test whether circSAMD4A can induce preadipocyte differentiation. It has been observed that some circRNAs can act as sponges for miRNAs [51], and circSAMD4A has been demonstrated to be a sponge of miR-138-5p since an internal ribosome entry site (IRES) was found in the structure of circSAMD4A [33]. Poor prognosis in obesity patients has been related to the overexpression of circSAMD4A; nevertheless, the knockdown of circSAMD4A can suppress the preadipocyte differentiation process by reducing miR-138-5p functionality. Furthermore, EZH2 was identified as the main target of miR-138-5p. EZH2 is a polycomb-group protein that is involved in suppressing gene transcription [52]. The expression of EZH2 was high in adipocytes from obese patients, which also had an increased expression of circSAMD4A. miR-138-5p upregulation significantly reduced EZH2 expression when circSAMD4A was overexpressed. Hence, it has been demonstrated that the circSAMD4A-miR-138-5p-EZH2 axis affects preadipocyte differentiation in obese patients and could be used as a therapeutic tool to treat obesity [33].

Some chemical compounds present in food can interact with miRNAs to prevent or alleviate obesity. For example, Du and colleagues in 2020 [36] hypothesized that in mice, betaine, a natural compound commonly present in beet, shellfish, spinach, Swiss chard, and other leafy green vegetables [36], can interact with gut microbiota-derived miR-378a and improve obesity since the miR-378a family acts as a regulator of numerous metabolic pathways, including autophagy and mitochondrial metabolism [53]. Likewise, more recently, Chen and collaborators (2022) [47] investigated the response of miR-143 in the presence of betaine in lipid metabolism. In wild-type mouse liver, it was observed that the expression of miR-143 was downregulated when betaine was supplemented. Also, the supplementation with betaine and miR-143 knockout caused obesity attenuation due to the repression of the glycoprotein nonmetastatic melanoma protein B (GPNMB), which has been reported to promote lipogenesis in white adipose tissue (WAT) and intensify diet-induced obesity [47,54]. Furthermore, fatty acid synthase (FASN) was upregulated when miR-143 was knocked out; nevertheless, FASN was observed to be downregulated when miR-143 knockout was supplemented with betaine. These mechanisms ultimately lead to a relevant reduction in fat proportion in the body weight as well as better energy consumption. Hence, miR-143 knockout, supplemented with betaine, could be a novel therapy for obesity management [47].

Gan et al. (2020) [44] investigated the molecular impact of genistein (4,5,7-trihydroxyisoflavone) on obesity using 3T3-L1 preadipocytes and obese mice as models. Genistein is an isoflavone found mainly in soybeans and soy products, such as tofu, tempeh, and miso, and in smaller amounts in chickpeas, alfalfa, or red clover [55]. Genistein is widely used in treating cancer, menopausal osteoporosis, inflammation, and depression. It has been noticed that genistein can regulate miRNAs, promoting antitumor, antidepressant, and antioxidant effects [44]. Previous studies have shown that miR-222, which is significantly associated with adipogenesis and obesity, could be inhibited by genistein in PC-3, MCF-7, and U87-MG cells [44]. In 3T3-L1 preadipocytes, genistein also inhibited the expression of miR-222 and promoted lipid decomposition. They also reported that genistein can potentially alleviate the expression of inflammatory cytokines TNFα and IL-6 associated with insulin resistance and inflammation in AT mice induced by a HFD.

Exosomal miRNAs have also been identified as potential tools for preventing obesity. Exosomes, small extracellular vesicles, may participate in the pathogenesis of obesity by acting as communication mediators [56,57]. Feng and collaborators (2022) analyzed the role of exosomal miR-452 and miR-4713 in obesity developed by children. Results revealed that both miRNAs were substantially downregulated in AT relative to normal tissue. The Neuropeptide Y Receptor Y1 (NPY1R), which was upregulated in AT, was identified as a prime target of both miR-452 and miR-4713 [37]. NPY1R is known as the appetite-promoting receptor, while the Neuropeptide Y stimulation of adipocytes is related to cell proliferation and mitogenesis [58]. Experimental work involving food deprivation showed a relevant reduction in the expression of NPY1R in the hypothalamic regions of rats [59]. Moreover, two novel pathways were found to be associated with NPY1R. In the first one, the transcription factors HEY1 and TCF1 showed a positive correlation with NPY1R by acting on miR-4713, while the other pathway involves GATA3, whose expression is reduced in obese patients, leading to the downregulation of miR-452 and the upregulation of NPY1R. Therefore, elucidating the aforementioned pathways is relevant to understanding how they can be used as therapeutic tools to treat obesity in children.

Similarly, Huang and colleagues (2022) explored the role of exosomal miR-122 in adipogenesis and obesity. In AT-derived exosomes (Exo-AT), it was noticed that miR-122 is overexpressed, promoting adipogenesis. miR-122 directly targets the vitamin D3 receptor (VDR), and VDR negatively regulates sterol regulatory element-binding transcription factor 1 (SREBF1) [38]. SREBF1 is a transcriptional factor related to lipid synthesis, regulating the homeostasis of cholesterol and fatty acids [60], and it was identified as a potential tool for treating obesity-related metabolic syndrome since SREBF1 inhibition can suppress lipogenesis. On the other hand, Exo-AT could increase miR-122 levels, inducing adipogenesis and modifying adipocytes’ morphologies. It was demonstrated that the inhibition of miR-122 by the injection of antagomiR-122 relieves adipogenesis in HFD-fed mice. Hence, the miR-122/VDR/SREBF1 axis might be upregulated by Exo-AT, promoting lipogenesis and speeding the development and progression of obesity, since miR-122 contributes to adipogenesis by inhibiting VDR and activating the SREBF1 signaling pathway [38]. Some other epigenetic factors that can promote the development of obesity are associated with exposure to chemicals that are not naturally occurring, such as Organophosphorus flame retardants (OPFRs), which can interact with miRNAs. OPFRs are widely used in several industries, including plastic and, more specifically, in food packaging [39,61]. Approximately 2-ethylhexyl diphenyl phosphate (EHDPP) is a type of OPFR used in food packaging with significant cytotoxic effects [62]. In this matter, Yue and collaborators (2023) recently discovered that miR-155-5p can contribute to the adipogenic activity of EHDPP in 3T3-L1 preadipocytes [39]. They observed that miR-155-5p was downregulated in 3T3-L1 preadipocytes exposed to EHDPP. C/EBPβ is a target gene of miR-155-5p, and it regulates the expression of diverse inflammatory factors such as IL-6 or IL-8. As well, C/EBPβ interacts with the C/EBP binding elements in the PPARγ promoters and activates its expression via miR-155-5p downregulation. Therefore, it was concluded that miR-155-5p plays a crucial role in mediating EHDPP adipogenic activity [39].

Li et al. (2022) [42] examined the mechanism by which miR-122-5p regulates cytokine expression in adipocytes during childhood obesity. They found that the expression of miR-122-5p was substantially low in Simpson–Golabi–Behmel syndrome (SGBS) preadipocytes of the cocultured system or treated with a macrophage-conditioned medium (MacCM). Moreover, when miR-122-5p mimics were applied, the mRNA and protein levels of chemokines and proinflammatory cytokines, such as interleukin 6 (IL-6), interleukin 8 (IL-8), and monocyte chemoattractant protein 1 (MCP-1) were notably decreased, indicating that upregulated miR-122-5p can substantially suppress cytokine expression in SGBS adipocytes. Interestingly, miR-122-5p overexpression in SGBS adipocytes significantly downregulates CPEB1 protein expression, elucidating that CPEB1 is a direct target of this miRNA. CPEB1 is involved in several biological mechanisms, such as apoptosis, differentiation, and epithelial-to-mesenchymal transition. In SGBG adipocytes, the overexpression of CPEB1 could hinder the inhibitory effect of miR-122-5p mimics on cytokine expression. These results suggest that miR-122-5p can act as an anti-inflammatory molecule in childhood obesity; nonetheless, further investigation is still needed to precisely elucidate the mechanism by which CPEB1 facilitates cytokine secretion [42].

Juiz-Valiña et al. (2022) [45] analyzed the expression pattern of the miR-19 family in the adipogenesis-induced 3T3-L1 cell line (1, 2, 4, and 6 days post-induction) and compared the results with those of the undifferentiated cells. Results revealed that the expression of both miR-19a and miR-19b is hindered during the differentiation process. Subsequently, to elucidate the effects of both miR-19a and miR-19b, they artificially overexpressed them in 3T3-L1 preadipocyte cells and noticed a drastic downregulation of adipogenic/lipogenic genes such as Pparg, Adipoq, Cebpa, and Fasn during adipogenesis [45].

Neurological control is one of the most relevant aspects of obesity. Inside the brain, there is a crucial region that regulates energy homeostasis, known as the arcuate hypothalamic nucleus (ARH), which is comprised of diverse types of cells that mediate metabolic functions, like the melanocortin system [43]. The two main neuronal populations express bioactive peptides with antagonistic roles: one is appetite-inducing (orexigenic), and the other is appetite-suppressing (anorexigenic) [43]. The anorexigenic neuronal population expresses Tbx3 and POMC, which signal to diverse hypothalamic and extra-hypothalamic structures to regulate energy expenditure and food intake [43]. In mice, POMC-dependent overexpression of the PI3K-Akt-mTOR pathway triggers hyperpolarization of POMC neurons, which results in obesity. Furthermore, in adult ARH, the expression of Dicer, a key nuclease for miRNA maturation, is crucial for energy homeostasis [43]. With the aim of exploring the metabolic roles of miRNAs in defined cellular populations within the mature hypothalamus, Ma et al. (2022) conducted a study [43], and they noted that depletion of miR-29-b-3p, miR-103a-3p, and miR-29a-3p significantly increased S6 ribosomal protein (pS6), a downstream target of the PI3K-Akt-mTOR pathway, and thus, the miR-29 family might block the overactivation of the pathway. Moreover, these miRNAs were found to be overexpressed in the mouse hypothalamus, with miR-29a-3p being highly abundant in POMC lineage-derived neurons and other cells in the ARH of adult mice. Through the Dicer1 knockout, they injected miR-29a-3p mimics into mice and observed remarkably reduced body and fat pad weights and food intake. This suggests that when miR-29a-3p is delivered in adulthood, it could diminish hyperphagic obesity. Additionally, downregulation of miR-29a-3p in ARH led to mild and transient weight gain in male mice, while in female mice, a late-onset weight gain and fat tissue hypertrophy were noticed, indicating the crucial role of miR-29a in energy homeostasis in ARH throughout adulthood. POMC-restricted depletion of the miR-29 family resulted in late-onset obesity, fat pad enlargement, and insulin resistance in aged female mice, while in males, it caused mild and transient but significant weight gain. Interestingly, several weeks before the appearance of the weight phenotype, young mice showed impaired insulin sensitivity; these results imply that miR-29a is also crucial for normal metabolic balance and is age-independent. Moreover, the authors investigated the putative targets of miR-29a-3p and found that Nras was the only gene in the PI3K-Akt pathway that was significantly downregulated in ARH when miR-29a-3p was overexpressed. Nras, a highly conserved GTPase and a strong activator of the PI3K-Akt pathway, was validated as a target of the miR-29 family in mice and humans. When miR-29 was suppressed, mice showed higher early-onset food intake, late-onset obesity, increased insulin resistance, and hypertrophy of adipocytes, while simultaneous deletion of Nras remarkably attenuated this phenotype. Overall, their investigation elucidated that in mature neurons from the POMC lineage, miR-29a can protect against insulin resistance, decreased energy expenditure, hyperphagia, and obesity [43].

One of the main triggers for obesity development is adipocyte apoptosis, leading to macrophage infiltration into AT and, consequently, inflammation. In this regard, Liu and collaborators [46,63] studied the role of the differential expression of miR-27a in human obesity. miR-27a was found to be downregulated in serum and the AT of both obese mice and humans, inducing apoptosis in adipocytes [46]. PPAR-γ has been identified as the main target of miR-27a, leading to adipocyte differentiation and repression [64]. Furthermore, when 3T3-L1 adipocytes were treated with miR-27a mimics and TNF-α, apoptosis was prevented in preadipocytes, concluding that the overexpression of miR-27a has an antiapoptotic effect. Hence, miR-27a can be a potential novel target for the treatment of obesity and obesity-related metabolic ailments [46].

Brown adipose tissue (BAT) has the potential to prevent and protect against obesity since it can dissipate chemical energy in the form of heat [48]. Lou and colleagues [48] identified miR-22 as a regulator of thermogenesis control in BAT, as it activates mTORC1 signaling in BAT. Also, miR-22 was found to be upregulated in BAT, targeting *Hif1an*, which is an inhibitor of glycolysis promotion and thermogenesis via the inhibition of Hif1α. Furthermore, Hu and collaborators [49] observed that the inhibition of miR-22 in human and mouse livers can reduce fat accumulation by inducing FGFR1, a novel target of miR-22. The deficiency of FGFR1 has been related to the termination of the intracellular transduction of FGF21 signaling, causing energy expenditure reduction and a decrease in fatty acid oxidation [49]. Due to the promissory results on the inhibition of miR-22, this miRNA could be a potential novel therapeutic tool for treating metabolic diseases, most relevantly obesity, due to its crucial role in fatty acid oxidation.

It has also been noticed that the expression of miRNAs varies depending on the stages of obesity pathogenesis and correlates to different biological mechanisms [65]. For example, in murine, alteration in miR-30a-5p, miR-133a-5p, and miR-107-5p levels was observed in the early stages of obesity, leading to disturbances in adipokines, insulin, and glucose levels [65,66]. As well, low miR-21 expression in the early stages of the disease correlates to the proliferation of adipocyte precursors, while high expression of miR-21 at the latter stages aids in enhanced adipogenic differentiation [66]. The aforementioned findings lead to the conclusion that the expression profile of miRNAs, as well as their role in the disease’s biological mechanisms, vary over time and are related to the stage in which obesity is present.

Additionally, it has been elucidated that in obesity, the titer of glucagon-like peptide-1 (GLP-1) declines, and this can lead to metabolic syndrome [50]. miR-194 has been identified as a regulator of GLP-1 since, in the plasma and ileum tissue of obese mice, GLP-1 was underexpressed while miR-194 was upregulated. In obese mice, the overexpression of miR-194 and the inhibition of GLP-1 expression led to mitochondrial dysfunction and aggravated cardiac injury [50]. Hence, miR-194 is an important biomarker in the expression of GLP-1 and in the interaction of obesity and its inherent biological mechanisms.

## 3. Roles of microRNAs in Obesity-Derived Ailments

miRNAs are associated with a wide variety of biological processes allied with obesity development, as well as other obesity-associated disorders. Liu, T., and colleagues (2019) discovered that the exosomal miR-29a is linked to obesity-associated insulin resistance, a precursor of the development of T2D [35], while AT macrophages (ATMs) are one of the main components of the obesity-induced tissue inflammatory state and can modulate processes such as insulin resistance [67]. It has been observed that in obese ATM-derived exosomes (both in vitro and in vivo), miR-29a is overexpressed when compared to lean subjects, and it can be transferred into hepatocytes, myocytes, and adipocytes, resulting in the development of insulin resistance. PPAR-d has been identified as the prime target of miR-29a, which mediates insulin signaling [35], while GW501516, the agonist of PPAR-d, can rescue the insulin resistance promoted by miR-29a. miR-29a has also been related to the mediation of glucolipid metabolism in adipocytes [68], and in obesity, ATM levels are increased, being correlated with adiposity [69]. Hence, this discovery could be promising for treating diseases associated with obesity, such as T2D.

Gjorgjieva and collaborators (2020) [41] investigated the relationship between metabolomic alterations in obesity-related non-alcoholic fatty liver disease (NAFLD) and miR-22-3p. In a small cohort of human liver biopsies from obese patients with steatosis, they observed that hepatic miR-22-3p was significantly underexpressed. Additionally, free fatty acid exposure to mouse primary hepatocytes (MPH), SK-Hep1 cells, or human hepatocytes (HPH) + inflammatory mediators (TNFα) also downregulate miR-22-3p. Moreover, in HFD-fed mice, the deletion of miR-22 induced higher and faster weight gain and upregulation of Alanine transaminase (ALAT), indicating hepatocellular damage. Likewise, in EWAT, the absence of miR-22 increased the protein levels of diverse enzymes involved in lipid synthesis, such as fatty acid synthase (FAS), acetyl CoA carboxylase (ACC), and stearoyl-CoA desaturase 1 (SCD1). After feeding mice with HFD for 12 weeks, the knockout of miR-22 resulted in severe hepatomegaly and extended hepatic steatosis. Furthermore, 54 potential miR-22-3p target candidates, of which 30 are associated with lipid/glucose metabolism, lipogenesis, glycolysis, and lipid catabolism trafficking, were identified. Additionally, CD36 and other lipid transporters, such as fatty acid transport proteins 1 and 2 (Fatp1 and Fatp2), were significantly upregulated. Although mRNA levels of FAS, ACC, and Scd1 remain unaffected, their protein expression was enhanced, suggesting translational blockade of specific transcripts by miR-22 and miR-22-dependent degradation. The expression of key factors associated with glycolysis, such as enolase 1 (Eno1), sedoheptulokinase (Shpk), glucokinase (Gck), pyruvate kinase M2 (Pkm2), and phosphofructokinase, liver type (Pfkl), was augmented when miR-22 was absent, indicating that miR-22 deficiency is related to the expression of critical factors that promote hepatic steatosis development in obesogenic conditions. Finally, in human hepatic cancer cell lines (Huh7, HepaRG, and HepG2), miR-22-3p displayed low expression levels, and for Huh7 cells, it prompts a classical Warburg-like metabolic switch from mitochondrial respiration to glycolysis. Altogether, their study highlights the role of miR-22-3p as a crucial regulator in lipid and glucose metabolism, exerting differential effects in specific organs and transformed hepatic cancer cells [41].

## 4. miRNA-Based Therapeutic Strategies in Obesity

Several therapeutic strategies that might even involve surgical intervention are viable options for the treatment of obesity, such as bariatric surgery. It has been observed that bariatric surgery has a significant influence on the expression of several miRNAs. Atkin and collaborators [70] reported that seven different miRNAs (miR-7-5p, let-7f-5p, miR-15b-5p, let-7i-5p, miR-320c, miR-205-5p, and miR-335-5p) had significant changes after 21 days of the surgical intervention. miR-7-5p and let-7f-5p showed expression in the pituitary, hence contributing to the control of adrenal and thyroid hormone release. Since let-7i-5p was observed to be altered after bariatric surgery, it has been related to the inhibition of beige adipocyte function and inhibiting the stimulation of brown thermogenic adipose tissue, suggesting that bariatric surgery aids in the accumulation of brown thermogenic fat. Likewise, the increased expression of miR-320c after bariatric surgery triggers adipocyte differentiation and accelerates the formation of mature adipocytes in vitro by targeting SOX4, FOXM, and FOXQ1 transcripts. Both miR-205-5p and miR-355-5p have been associated with cellular proliferation as well as several types of cancer [70]. Due to the expression alteration of obesity-related miRNAs, it can be concluded that bariatric surgery can be used to monitor the results of the intervention.

Other strategies for obesity alleviation include aerobic exercise training, which has been related to a change in miRNA expression profile [71,72,73]. Zhou and colleagues [73] observed that after cardiopulmonary exercise training (CPET) and 1 h acute exercise training (AET), the expression level of miR-1, miR-20a, miR-21, miR-126, miR-133a, miR-133b, miR-146, miR-155, miR-208a, miR-208b, miR-210, miR-221, miR-222, miR-328, miR-378, miR-499, and miR-940a had changed on their expression profiles. Among these miRNAs, serum miR-20a was underexpressed after CPET. miR-20a targets TNFSF15, which hinders the inhibition of angiogenesis. As well, serum miR-21 was overexpressed after AET, and it was elucidated that it promotes angiogenesis and has anti-apoptotic and anti-inflammatory effects [73]. Moreover, miRNAs, such as miR-1, miR-208a, and miR-16, were found to be downregulated after exercise training, increasing the expression of their targets (NCX1, MED13, and VEGF16), respectively, which have a protective role in obesity, while miR-126, miR-214, and miR-29c were noticed to be upregulated, decreasing the expression of their pathogenic targets PI3KR2, PTEN, and COLI, respectively (Table 2) [74].

## 5. Discussion and Conclusions

Multiple miRNAs are found to be associated with obesity and obesity-associated ailments, such as diabetes or NAFLD. In obesity, preadipocyte differentiation and proliferation, lipid metabolism, and adipogenesis are the processes that are affected the most by miRNAs, while insulin resistance seems to be a biological mechanism that is commonly regulated by miRNAs in obese individuals.

Chemical compounds present in the diet, such as genistein or betaine, can modulate the expression of miRNAs in obesity and related ailments, contributing to biological mechanisms such as lipolysis, inflammation, or the improvement of the gut microbiota. On the other hand, non-naturally occurring molecules used in some stages of food production can induce obesity by interacting with miRNAs, regulating processes such as inflammation and adipogenic activity.

Exosomal miRNAs are also substantially involved in multiple biological processes associated with obesity development or related disorders, such as adipocyte proliferation, mitogenesis, lipogenesis suppression, insulin resistance development, or even glucose tolerance. Moreover, it was observed that miRNA expression has a crucial role in some neurological mechanisms, such as the hyperpolarization of POMC neurons that leads to obesity. Furthermore, therapeutic strategies such as bariatric surgery or aerobic exercise have a strong influence on the miRNA expression profile in obesity, which helps in understanding the efficiency of these treatments.

Nonetheless, with the reviewed literature, it is concluded that relevant research still needs to be conducted to understand precisely how miRNA activity is involved in the processes underlying obesity development, as well as how these tiny molecules could be utilized for disease management.

## Figures and Tables

**Table 1:** List of miRNAs involved (relevant miRNAs involved in the presented metabolic pathways are only partially listed) in developing obesity and obesity-related ailments, along with their observed differential expression, targets, biological mechanisms, and samples.

Associated miRNAs	Target	Altered Biological Mechanism	Source/Sample	Reference
miR-29a ↑	PPAR-d	Promotion of insulin resistance	ATM-derived exosomes	[35]
miR-138-5p ↑	EZH2	Preadipocyte differentiation	AT	[33]
miR-378a ↑	YY1	Regulation of lipid and glucose metabolism Inhibition of lipogenesis	WAT and liver tissue	[36]
miR-4713 ↓	NPY1R	Adipocyte proliferation and mitogenesis	AT	[37]
miR-452 ↓				
miR-122 ↑	VDR	Lipid synthesis Homeostasis of cholesterol and fatty acids Adipogenesis	Exo-AT	[38]
miR-155-5p ↓	C/EBPβ	Adipogenesis Expression of IL-6 and IL-8	3T3-L1 preadipocytes	[39]
miR-129-5p ↑	ATG7	Adipocyte differentiation	AT in obese mice	[40]
miR-22-3p ↓	CD36, Fatp1, Fatp2, FAS, ACC, Scd1, Eno1, Shpk, Gck, Pkm2, Pfkl	Regulation of lipid and glucose metabolism	Human liver biopsies of obese patients	[41]
miR-122-5p ↓	CPEB1	Regulation of chemokines and proinflammatory cytokines	SGBS preadipocytes	[42]
miR-29a-3p ↑	Nras	Protection against insulin resistance and obesity Decreased energy expenditure	Brain tissue	[43]
miR-181d ↑	ANGPTL3	Regulation of lipid metabolism	Human plasma	[32]
miR-222 ↑	BTG2 adipor1	Regulation of lipid metabolism	3T3-L1 preadipocytes and in vivo	[44]
miR-19a ↑	Pparg Cebpa Adipoq Fasn	Regulation of adipogenesis and lipid accumulation	3T3-L1 cells	[45]
miR-19b ↑				
miR-27a ↓	PPAR-γ	Adipocyte differentiation and repression	Serum and AT	[46]
miR-143 ↓	GPNMB	Regulation of lipogenesis	WAT	[47]
miR-22 ↑	Hif1an	Regulation of glycolysis and thermogenesis	BAT	[48]
miR-22 ↓	FGFR1	Regulation of fatty acid oxidation	BAT	[49]
miR-194 ↑	GLP-1	Mitochondrial dysfunction and cardiac injury	Plasma and ileum tissue of mice	[50]

↑ Upregulated (overexpressed) miRNA; ↓ Downregulated (underexpressed) miRNA.

**Table 2:** Relationship between therapeutic strategies in obesity and miRNAs, as well as their biological implications for the treatment of obesity.

Therapeutic Strategy	Altered miRNAs	Implications of the Treatment in Obesity	Reference
Bariatric surgery	miR-7-5p let-7f-5p miR-15b-5p let-7i-5p miR-320x miR-205-5p miR-335-5p	Enhancement of adipocyte differentiationInhibition of beige adipocyte function Aid in the accumulation of brown thermogenic fat	[70]
CPET and AET	miR-1 miR-20a miR-21 miR-126 miR-133a miR-133b miR-146 miR-155 miR-208a miR-208b miR-210 miR-221 miR-222 miR-328 miR-378 miR-499 miR-940a	Promotion of angiogenesis Anti-apoptotic effects Anti-inflammatory effects	[71,72,73,74]

## Data Availability

Not applicable.

## References

[1] Guerreiro V.A., Carvalho D., Freitas P. (2022). Obesity, Adipose Tissue, and Inflammation Answered in Questions. J. Obes..

[2] Caballero B. (2019). Humans against Obesity: Who Will Win?. Adv. Nutr..

[3] Müller T.D., Blüher M., Tschöp M.H., DiMarchi R.D. (2022). Anti-Obesity Drug Discovery: Advances and Challenges. Nat. Rev. Drug Discov..

[4] Yu W., Rohli K.E., Yang S., Jia P. (2021). Impact of Obesity on COVID-19 Patients. J. Diabetes Complicat..

[5] Oussaada S.M., van Galen K.A., Cooiman M.I., Kleinendorst L., Hazebroek E.J., van Haelst M.M., ter Horst K.W., Serlie M.J. (2019). The Pathogenesis of Obesity. Metabolism..

[6] Morales I. (2022). Brain Regulation of Hunger and Motivation: The Case for Integrating Homeostatic and Hedonic Concepts and Its Implications for Obesity and Addiction. Appetite.

[7] Miller J. (2023). Genetic Obesity—Causes and Treatments. Pediatr. Ann..

[8] Thaker V. (2017). V Genetic and Epigenetic Causes of Obesity. Adolesc. Med. State Art Rev..

[9] Mahmoud R., Kimonis V., Butler M.G. (2022). Genetics of Obesity in Humans: A Clinical Review. Int. J. Mol. Sci..

[10] Baxter J., Armijo P.R., Flores L., Krause C., Samreen S., Tanner T. (2019). Updates on Monogenic Obesity in a Multifactorial Disease. Obes. Surg..

[11] Carvalho L.M.L., D’Angelo C.S., Villela D., da Costa S.S., de Lima Jorge A.A., da Silva I.T., de Oliveira Scliar M., Chaves L.D., Krepischi A.C.V., Koiffmann C.P. (2022). Genetic Investigation of Syndromic Forms of Obesity. Int. J. Obes..

[12] Loos R.J.F., Yeo G.S.H. (2022). The Genetics of Obesity: From Discovery to Biology. Nat. Rev. Genet..

[13] Wu F.Y., Yin R.X. (2022). Recent Progress in Epigenetics of Obesity. Diabetol. Metab. Syndr..

[14] Ruiz-Manriquez L.M., Villarreal-Garza C., Benavides-Aguilar J.A., Torres-Copado A., Isidoro-Sánchez J., Estrada-Meza C., Arvizu-Espinosa M.G., Paul S., Cuevas-Diaz Duran R. (2023). Exploring the Potential Role of Circulating MicroRNAs as Biomarkers for Predicting Clinical Response to Neoadjuvant Therapy in Breast Cancer. Int. J. Mol. Sci..

[15] Benavides-Aguilar J.A., Morales-Rodríguez J.I., Ambriz-González H., Ruiz-Manriquez L.M., Banerjee A., Pathak S., Duttaroy A.K., Paul S. (2023). The Regulatory Role of MicroRNAs in Common Eye Diseases: A Brief Review. Front. Genet..

[16] Kargutkar N., Hariharan P., Nadkarni A. (2023). Dynamic Interplay of MicroRNA in Diseases and Therapeutic. Clin. Genet..

[17] Ledesma-Pacheco S.J., Uriostegui-Pena A.G., Rodriguez-Jacinto E., Gomez-Hernandez E., Estrada-Meza C., Banerjee A., Pathak S., Ruiz-Manriquez L.M., Duttaroy A.K., Paul S. (2023). Regulatory Mechanisms of MicroRNAs in Endocrine Disorders and Their Therapeutic Potential. Front. Genet..

[18] Guo J., Yang P., Li Y.F., Tang J.F., He Z.X., Yu S.G., Yin H.Y. (2023). MicroRNA: Crucial Modulator in Purinergic Signalling Involved Diseases. Purinergic Signal..

[19] Ruiz-Manriquez L.M., Carrasco-Morales O., Sanchez Z.E.A., Osorio-Perez S.M., Estrada-Meza C., Pathak S., Banerjee A., Bandyopadhyay A., Duttaroy A.K., Paul S. (2022). MicroRNA-Mediated Regulation of Key Signaling Pathways in Hepatocellular Carcinoma: A Mechanistic Insight. Front. Genet..

[20] Samad A.F.A., Kamaroddin M.F. (2023). Innovative Approaches in Transforming MicroRNAs into Therapeutic Tools. WIREs RNA.

[21] Bayraktar E., Bayraktar R., Oztatlici H., Lopez-Berestein G., Amero P., Rodriguez-Aguayo C. (2023). Targeting MiRNAs and Other Non-Coding RNAs as a Therapeutic Approach: An Update. Non-Coding RNA.

[22] Smolarz B., Durczyński A., Romanowicz H., Szyłło K., Hogendorf P. (2022). MiRNAs in Cancer (Review of Literature). Int. J. Mol. Sci..

[23] Iacomino G. (2023). MiRNAs: The Road from Bench to Bedside. Genes.

[24] Matsuyama H., Suzuki H.I. (2020). Systems and Synthetic MicroRNA Biology: From Biogenesis to Disease Pathogenesis. Int. J. Mol. Sci..

[25] Rani V., Sengar R.S. (2022). Biogenesis and Mechanisms of MicroRNA-Mediated Gene Regulation. Biotechnol. Bioeng..

[26] Pelletier D., Rivera B., Fabian M.R., Foulkes W.D. (2023). MiRNA Biogenesis and Inherited Disorders: Clinico-Molecular Insights. Trends Genet..

[27] Vishnoi A., Rani S., Rani S. (2017). MiRNA Biogenesis and Regulation of Diseases: An Overview. MicroRNA Profiling: Methods and Protocols.

[28] Ruiz-Manriquez L.M., Estrada-Meza C., Benavides-Aguilar J.A., Ledesma-Pacheco S.J., Torres-Copado A., Serrano-Cano F.I., Bandyopadhyay A., Pathak S., Chakraborty S., Srivastava A. (2022). Phytochemicals Mediated Modulation of MicroRNAs and Long Non-Coding RNAs in Cancer Prevention and Therapy. Phyther. Res..

[29] Bravo-Vázquez L.A., Frías-Reid N., Ramos-Delgado A.G., Osorio-Pérez S.M., Zlotnik-Chávez H.R., Pathak S., Banerjee A., Bandyopadhyay A., Duttaroy A.K., Paul S. (2023). MicroRNAs and Long Non-Coding RNAs in Pancreatic Cancer: From Epigenetics to Potential Clinical Applications. Transl. Oncol..

[30] Paul S., Ruiz-Manriquez L.M., Ledesma-Pacheco S.J., Benavides-Aguilar J.A., Torres-Copado A., Morales-Rodríguez J.I., De Donato M., Srivastava A. (2021). Roles of MicroRNAs in Chronic Pediatric Diseases and Their Use as Potential Biomarkers: A Review. Arch. Biochem. Biophys..

[31] Elkhawaga S.Y., Ismail A., Elsakka E.G.E., Doghish A.S., Elkady M.A., El-Mahdy H.A. (2023). MiRNAs as Cornerstones in Adipogenesis and Obesity. Life Sci..

[32] Abu-Farha M., Cherian P., Al-Khairi I., Nizam R., Alkandari A., Arefanian H., Tuomilehto J., Al-Mulla F., Abubaker J. (2019). Reduced MiR-181d Level in Obesity and Its Role in Lipid Metabolism via Regulation of ANGPTL3. Sci. Rep..

[33] Liu Y., Liu H., Li Y., Mao R., Yang H., Zhang Y., Zhang Y., Guo P., Zhan D., Zhang T. (2020). Circular RNA SAMD4A Controls Adipogenesis in Obesity through the MiR-138-5p/EZH2 Axis. Theranostics.

[34] Liu J., Wang H., Zeng D., Xiong J., Luo J., Chen X., Chen T., Xi Q., Sun J., Ren X. (2023). The Novel Importance of MiR-143 in Obesity Regulation. Int. J. Obes..

[35] Liu T., Sun Y.C., Cheng P., Shao H.G. (2019). Adipose Tissue Macrophage-Derived Exosomal MiR-29a Regulates Obesity-Associated Insulin Resistance. Biochem. Biophys. Res. Commun..

[36] Du J., Zhang P., Luo J., Shen L., Zhang S., Gu H., He J., Wang L., Zhao X., Gan M. (2021). Dietary Betaine Prevents Obesity through Gut Microbiota-Drived MicroRNA-378a Family. Gut Microbes.

[37] Feng X., Ding Y., Zhou M., Song N., Ding Y. (2022). Integrative Analysis of Exosomal MiR-452 and MiR-4713 Downregulating NPY1R for the Prevention of Childhood Obesity. Dis. Markers.

[38] Huang X.Y., Chen J.X., Ren Y., Fan L.C., Xiang W., He X.J. (2022). Exosomal MiR-122 Promotes Adipogenesis and Aggravates Obesity through the VDR/SREBF1 Axis. Obesity.

[39] Yue J., Sun C., Tang J., Zhang Q., Lou M., Sun H., Zhang L. (2023). Downregulation of MiRNA-155–5p Contributes to the Adipogenic Activity of 2-Ethylhexyl Diphenyl Phosphate in 3T3-L1 Preadipocytes. Toxicology.

[40] Fu X., Jin L., Han L., Yuan Y., Mu Q., Wang H., Yang J., Ning G., Zhou D., Zhang Z. (2019). MiR-129-5p Inhibits Adipogenesis through Autophagy and May Be a Potential Biomarker for Obesity. Int. J. Endocrinol..

[41] Gjorgjieva M., Sobolewski C., Ay A.S., Abegg D., de Sousa M.C., Portius D., Berthou F., Fournier M., Maeder C., Rantakari P. (2020). Genetic Ablation of MiR-22 Fosters Diet-Induced Obesity and NAFLD Development. J. Pers. Med..

[42] Li D., Chen J., Yun C., Li X., Huang Z. (2022). MiR-122–5p Regulates the Pathogenesis of Childhood Obesity by Targeting CPEB1. Obes. Res. Clin. Pract..

[43] Ma Y., Murgia N., Liu Y., Li Z., Sirakawin C., Konovalov R., Kovzel N., Xu Y., Kang X., Tiwari A. (2022). Neuronal MiR-29a Protects from Obesity in Adult Mice. Mol. Metab..

[44] Gan M., Shen L., Wang S., Guo Z., Zheng T., Tan Y., Fan Y., Liu L., Chen L., Jiang A. (2020). Genistein Inhibits High Fat Diet-Induced Obesity through MiR-222 by Targeting BTG2 and Adipor1. Food Funct..

[45] Juiz-Valiña P., Varela-Rodríguez B.M., Outeiriño-Blanco E., García-Brao M.J., Mena E., Cordido F., Sangiao-Alvarellos S. (2022). MiR-19 Family Impairs Adipogenesis by the Downregulation of the PPARγ Transcriptional Network. Int. J. Mol. Sci..

[46] Liu L., Li D., Peng C., Gao R., Li X., Zhang L., Lv Q., Xiao X., Li Q. (2023). MicroRNA-27a, Downregulated in Human Obesity, Exerts an Antiapoptotic Function in Adipocytes. Endocr. J..

[47] Chang R.C., Joloya E.M., Li Z., Shoucri B.M., Shioda T., Blumberg B. (2023). MiR-223 Plays a Key Role in Obesogen-Enhanced Adipogenesis in Mesenchymal Stem Cells and in Transgenerational Obesity. Endocrinology.

[48] Lou P., Bi X., Tian Y., Li G., Kang Q., Lv C., Song Y., Xu J., Sheng X., Yang X. (2021). MiR-22 Modulates Brown Adipocyte Thermogenesis by Synergistically Activating the Glycolytic and MTORC1 Signaling Pathways. Theranostics.

[49] Hu Y., Liu H.X., Jena P.K., Sheng L., Ali M.R., Wan Y.J.Y. (2020). MiR-22 Inhibition Reduces Hepatic Steatosis via FGF21 and FGFR1 Induction. JHEP Rep..

[50] Wang J., Zhao D., Ding C.Z., Guo F., Wu L.N., Huang F.J., Liu Y.L., Zhao S.Y., Xin Y., Ma S.N. (2021). MicroRNA-194: A Novel Regulator of Glucagon-like Peptide-1 Synthesis in Intestinal L Cells. Cell Death Dis..

[51] Guo L., Jia L., Luo L., Xu X., Xiang Y., Ren Y., Ren D., Shen L., Liang T. (2022). Critical Roles of Circular RNA in Tumor Metastasis via Acting as a Sponge of MiRNA/IsomiR. Int. J. Mol. Sci..

[52] Wang H. (2022). Role of EZH2 in Adipogenesis and Obesity: Current State of the Art and Implications-A Review. Medicine.

[53] Machado I.F., Teodoro J.S., Palmeira C.M., Rolo A.P. (2020). MiR-378a: A New Emerging MicroRNA in Metabolism. Cell. Mol. Life Sci..

[54] Gong X.-M., Li Y.-F., Luo J., Wang J.-Q., Wei J., Wang J.-Q., Xiao T., Xie C., Hong J., Ning G. (2019). Gpnmb Secreted from Liver Promotes Lipogenesis in White Adipose Tissue and Aggravates Obesity and Insulin Resistance. Nat. Metab..

[55] Rasheed S., Rehman K., Shahid M., Suhail S., Akash M.S.H. (2022). Therapeutic Potentials of Genistein: New Insights and Perspectives. J. Food Biochem..

[56] Cabiati M., Randazzo E., Guiducci L., Falleni A., Cecchettini A., Casieri V., Federico G., Del Ry S. (2023). Evaluation of Exosomal Coding and Non-Coding RNA Signature in Obese Adolescents. Int. J. Mol. Sci..

[57] Ferrante S.C., Nadler E.P., Pillai D.K., Hubal M.J., Wang Z., Wang J.M., Gordish-Dressman H., Koeck E., Sevilla S., Wiles A.A. (2015). Adipocyte-Derived Exosomal MiRNAs: A Novel Mechanism for Obesity-Related Disease. Pediatr. Res..

[58] Wittrisch S., Klöting N., Mörl K., Chakaroun R., Blüher M., Beck-Sickinger A.G. (2020). NPY1R-Targeted Peptide-Mediated Delivery of a Dual PPARα/γ Agonist to Adipocytes Enhances Adipogenesis and Prevents Diabetes Progression. Mol. Metab..

[59] Wang C., Yang N., Wu S., Liu L., Sun X., Nie S. (2007). Difference of NPY and Its Receptor Gene Expressions between Obesity and Obesity-Resistant Rats in Response to High-Fat Diet. Horm. Metab. Res..

[60] Sun S., Cao X., Castro L.F.C., Monroig Ó., Gao J. (2021). A Network-Based Approach to Identify Protein Kinases Critical for Regulating Srebf1 in Lipid Deposition Causing Obesity. Funct. Integr. Genom..

[61] Du J., Li H., Xu S., Zhou Q., Jin M., Tang J. (2019). A Review of Organophosphorus Flame Retardants (OPFRs): Occurrence, Bioaccumulation, Toxicity, and Organism Exposure. Environ. Sci. Pollut. Res..

[62] Shen J., Zhang Y., Yu N., Crump D., Li J., Su H., Letcher R.J., Su G. (2019). Organophosphate Ester, 2-Ethylhexyl Diphenyl Phosphate (EHDPP), Elicits Cytotoxic and Transcriptomic Effects in Chicken Embryonic Hepatocytes and Its Biotransformation Profile Compared to Humans. Environ. Sci. Technol..

[63] Sprenkle N.T., Winn N.C., Bunn K.E., Zhao Y., Park D.J., Giese B.G., Karijolich J.J., Ansel K.M., Serezani C.H., Hasty A.H. (2023). The MiR-23-27-24 Clusters Drive Lipid-Associated Macrophage Proliferation in Obese Adipose Tissue. Cell Rep..

[64] Torres J.L., Usategui-Martín R., Hernández-Cosido L., Bernardo E., Manzanedo-Bueno L., Hernández-García I., Mateos-Díaz A.M., Rozo O., Matesanz N., Salete-Granado D. (2022). PPAR-γ Gene Expression in Human Adipose Tissue Is Associated with Weight Loss After Sleeve Gastrectomy. J. Gastrointest. Surg..

[65] Youssef E.M., Elfiky A.M., Banglysoliman, Abu-Shahba N., Elhefnawi M.M. (2020). Expression Profiling and Analysis of Some MiRNAs in Subcutaneous White Adipose Tissue during Development of Obesity. Genes Nutr..

[66] Abente E.J., Subramanian M., Ramachandran V., Najafi-Shoushtari S.H. (2016). MicroRNAs in Obesity-Associated Disorders. Arch. Biochem. Biophys..

[67] Ying W., Riopel M., Bandyopadhyay G., Dong Y., Birmingham A., Seo J.B., Ofrecio J.M., Wollam J., Hernandez-Carretero A., Fu W. (2017). Adipose Tissue Macrophage-Derived Exosomal MiRNAs Can Modulate in Vivo and in Vitro Insulin Sensitivity. Cell.

[68] Dooley J., Garcia-Perez J.E., Sreenivasan J., Schlenner S.M., Vangoitsenhoven R., Papadopoulou A.S., Tian L., Schonefeldt S., Serneels L., Deroose C. (2016). The MicroRNA-29 Family Dictates the Balance between Homeostatic and Pathological Glucose Handling in Diabetes and Obesity. Diabetes.

[69] Weisberg S.P., McCann D., Desai M., Rosenbaum M., Leibel R.L., Ferrante A.W. (2003). Obesity Is Associated with Macrophage Accumulation in Adipose Tissue. J. Clin. Investig..

[70] Atkin S.L., Ramachandran V., Yousri N.A., Benurwar M., Simper S.C., McKinlay R., Adams T.D., Najafi-Shoushtari S.H., Hunt S.C. (2019). Changes in Blood MicroRNA Expression and Early Metabolic Responsiveness 21 Days Following Bariatric Surgery. Front. Endocrinol..

[71] das Neves V.J., Fernandes T., Roque F.R., Soci U.P.R., Melo S.F.S., de Oliveira E.M. (2014). Exercise Training in Hypertension: Role of MicroRNAs. World J. Cardiol..

[72] Improta-Caria A.C., Vasques Nonaka C.K.V., Pereira C.S., Soares M.B.P., Macambira S.G., Souza B.S.d.F. (2018). Exercise Training-Induced Changes in MicroRNAs: Beneficial Regulatory Effects in Hypertension, Type 2 Diabetes, and Obesity. Int. J. Mol. Sci..

[73] Zhou Q., Shi C., Lv Y., Zhao C., Jiao Z., Wang T. (2020). Circulating MicroRNAs in Response to Exercise Training in Healthy Adults. Front. Genet..

[74] Improta-Caria A.C., Soci Ú.P., Rodrigues L.F., Fernandes T., Oliveira E.M. (2023). MicroRNAs Regulating Pathophysiological Processes in Obesity: The Impact of Exercise Training. Curr. Opin. Physiol..

